# Distinct Infection Forms of SARS-CoV-2 Among People Living With HIV

**DOI:** 10.21203/rs.3.rs-569883/v2

**Published:** 2021-07-19

**Authors:** Mengmeng Wu, Fangzhao Ming, Songjie Wu, Yanbin Liu, Xiaoxia Zhang, Wei Guo, Gifty Marly, Weiming Tang, Ke Liang

**Affiliations:** Zhongnan Hospital of Wuhan University; Wuchang District Center for Disease Control and Prevention; Zhongnan Hospital of Wuhan University; Zhongnan Hospital of Wuhan University; Zhongnan Hospital of Wuhan University; Zhongnan Hospital of Wuhan University; Nanjing Medical University; Southern Medical University; Zhongnan Hospital of Wuhan University

**Keywords:** SARS-CoV-2, People living with human immunodeficiency virus (PLWH), asymptomatic carrier, unapparent infector

## Abstract

**Background:**

People living with HIV (PLWH) are immunodeficient, it is vague if they are more susceptible to SARS-CoV-2 infection than HIV negative individuals.

**Methods:**

In this cross-sectional study, 857 PLWH and 1048 HIV negative individuals were enrolled from the Wuchang district in Wuhan, China. We compared the total rate of SARS-CoV-2 infection, the rate of COVID-19, asymptomatic carriers, and unapparent infectors in the two groups. The risk factors associated with SARS-CoV-2 infection among PLWH were explored.

**Results:**

Fourteen out of 857 (1.63%) PLWH were infected with SARS-CoV-2, while 68 of 1048 (6.49%) HIV negative individuals were infected. In PLWH, there were 6 confirmed COVID-19 (0.70%), 4 asymptomatic carriers (0.47%) and 4 unapparent infectors (0.47%). In the HIV negative group, the cases of COVID-19, asymptomatic carrier, and unapparent infector were 5 (0.48%), 0 (0.00%), and 63 (6.01%), respectively. After adjusting for age, gender, and chronic comorbidities, the rate of SARS-CoV-2 infection in PLWH was lower than that in HIV negative group (1.96% vs 5.74%, P = 0.001). The morbidity of COVID-19 was similar between the two groups (P = 0.107), but the rate of unapparent infection in PLWH was lower than that in the HIV negative group (0.54% vs 5.46%, P = 0.001). Older age (aOR = 4.50, 95%CI: 1.34–15.13, P = 0.015) and OIs (aOR = 9.59, 95%CI: 1.54–59.92, P = 0.016) were risk factors for SARS-CoV-2 infection among PLWH.

**Conclusions:**

PLWH has different infection forms of SARS-CoV-2 compared with the general population. Older age and OIs were considered to driving causes of SARS-CoV-2 infection among PLWH.

## Background

By May 21st, 2021, a total of 165,580,045 confirmed cases and 3,431,857 deaths had been reported globally since the coronavirus disease 2019 (COVID-19) outbreak.[1] Due to the immune deficiency caused by the human immunodeficiency virus (HIV), people living with HIV (PLWH) were thought to be more vulnerable to the severe acute respiratory syndrome coronavirus 2 (SARS-CoV-2).[2] However, the current evidence is still lacking and inconsistent, as some studies have indicated that the morbidity of COVID-19 in PLWH is not higher than in the general population.[3] In addition, most of the previous studies did not take asymptomatic carriers and unapparent infectors into consideration, and they are largely missed in the existing literature.[4, 5] Whether the evidence will be similar to the existing literature after considering these two groups of individuals are still vague.

This study aimed to further investigate the prevalence and associated risk factors of SARS-CoV-2 infection in PLWH and HIV negative individuals in Wuhan, China, the earliest epicenter of COVID-19.

## Methods

### Study design and participants recruitment

As an extension of our former work[6, 7], this cross-sectional study has consisted of two groups of the population that participated in the seroepidemiological survey of SARS-CoV-2 in Wuchang district, Wuhan. The investigation was proceeded from May 1, 2020, to May 31, 2020. All the participants (age ≥ 18 years old) were lived in the Wuchang district for at least 1 month from December 1, 2019, to April 8, 2020.

All PLWH who were managed by the Wuchang district center for disease control and prevention (CDC) were recruited. The participants who were tested positive for HIV have been reported to Wuchang CDC through the China National HIV/AIDS Comprehensive Response Information Management System (CRIMS).

For the general group, a two-stage cluster sampling method was used to recruit the study population. Selecting communities as primary sampling units (PSUs) at the first stage and families at the second stage. Overall, all communities were certainty PSUs and 11 communities were selected with probability proportional to the size sampling method. Within each community, 36 households were selected by systematic random sampling method and all members of the households were invited to participate in the study. If individuals of a certain age group were missing, we swallowed the sample randomly to ensure that the age structure of the sample was similar to the natural structure of the population.

### Data collection

For PLWH, demographic information includes age, gender, chronic comorbidities, the mode of HIV acquisition, antiretroviral (ARV) regimens, current opportunistic infections (OIs). ARV regimens were obtained from CRIMS. Basic information about HIV negative participants was collected through a questionnaire. All participants were inquired of COVID-19 history, and we double-checked the name and identification card number with that of recorded COVID-19 patients in the CDC information management systems. All SARS-CoV-2 infections are diagnosed according to the 8th edition of clinical practice guidelines for COVID-19 in China.[8] The total SARS-CoV-2 infection rate including the rate of COVID-19, asymptomatic carrier, and unapparent infector.

### Definitions

Chronic comorbidities include hypertension, diabetes, chronic respiratory disease, cancer, and any other chronic disease that has been diagnosed. The definition of OIs was referring to the guideline formulated by the U.S. Department of Health and Human Services (DHHS).[9] The Asymptomatic carrier is defined as a patient who does not have clinical manifestations of COVID-19 but the nucleic acid is positive. The unapparent infector is defined as a patient who does not have clinical manifestations and nucleic acid negative but serum antibody for SARS-CoV-2 was positive.

### Laboratory procedures

The CD4 + T lymphocyte count (CD4 count) and HIV viral load (HIV-VL) were detected for PLWH. All recruited general individuals received HIV antibody screening tests. Methods for laboratory confirmation of SARS-CoV-2 infection included: respiratory specimens SARS-CoV-2 real-time fluorescence Polymerase Chain Reaction (RT-PCR), serum SARS-CoV-2 IgM/IgG antibody colloidal gold test, and magnetic particle chemiluminescence (qualitative result). The detection kits were provided by Shengxiang Biotechnology Co., LTD, and Guangzhou Wanfu Biotech Co., LTD. The kits were approved by the China Food and Drug Administration (FDA). In this study, swab nucleic and serum antibodies (IgM/IgG) were detected for all subjects. All positive specimens (nucleic acid, IgM, or IgG positive) were sent to China CDC for confirmation.

### Statistical analysis

Continuous variables were expressed as means (SD) or median (interquartile range) and categorical variables were expressed as frequency and percent. Comparisons of continuous variables were assessed using the independent sample T-test or Wilcoxon rank-sum test, while categorical variables were assessed using the χ^2^ test or the Fisher exact test. We calculated the crude rate and 95% confidence interval (95% CI) of SARS-CoV-2 infection to estimated using the exact binomial distribution. Then we used a logistic regression model to calculate the adjusted rate and 95% CI of SARS-CoV-2 infection after adjusting for age, gender, and chronic comorbidities to compare the difference in SARS-CoV-2 infection rate between PLWH and the HIV negative group in the Wuchang district. Univariate and multivariable modified Poisson regression methods were used to explore the risk factors associated with PLWH co-infected with SARS-CoV-2.

Statistical significance was defined as a two-sided p-value of less than 0.05. All analyses were conducted using STATA version 13.0 (STATA Corporation, College Station, Texas) and IBM SPSS Statistics (Version 26.0) software.

## Results

### Participants enrolled in the study

Totally, 910 PLWH were managed in Wuchang CDC. But 2 were excluded because they were living abroad during the closure of Wuhan and 51 refused to participate in this study. The control group consisted of 1,100 HIV-negative individuals of the general population selected from residents living in the Wuchang district, and 52 refused to participate in the study. In total, 857 PLWH and 1048 HIV negative participants were enrolled in this study. The PLWH were younger than HIV negative subjects (P = 0.001). The PLWH were predominantly male (P = 0.001) and had fewer comorbidities than the HIV negative population (P = 0.001) (Table 1).

### SARS-CoV-2 infection between PLWH and HIV negative group

In PLWH, there were 6 confirmed COVID-19 (0.70%), 4 asymptomatic carriers (0.47%) and 4 unapparent infectors (0.47%). In HIV negative group, the number of confirmed COVID-19, asymptomatic carriers, and unapparent infectors were 5 (0.48%), 0 (0.00%), and 63 (6.01%), respectively. The crude SARS-CoV-2 infection rate was 1.63% (14/857) in PLWH and 6.49%(68/1048)in HIV negative group. After adjusting for age, gender, and chronic comorbidities by the logistic regression model, the adjusted rate of SARS-CoV-2 infection in PLWH (1.96%, 95%CI: 0.90–3.01) was lower than that in the HIV negative group (5.74%, 95%CI: 4.31–7.17) (P = 0.001). But the adjusted rate of COVID-19 was no difference between PLWH (1.10%, 95%CI: 0.11–2.10) and HIV negative group (0.37%, 95%CI: 0.04–0.69) (P = 0.107). The adjusted rate of unapparent infection in PLWH (0.54%, 95%CI: 0.00-1.07) were also lower than in HIV negative group (5.46%, 95%CI: 4.02–6.91) (P = 0.001) (Table 2).

### Comparison of the characteristics of SARS-CoV-2 infected and non-infected PLWH

PLWH infected with SARS-CoV-2 had older age than those who did not infect with SARS-CoV-2 (53.5 years vs 35.0 years, P = 0.005), and a higher rate of chronic comorbidities (P = 0.048). In addition, PLWH with OIs had a higher SARS-CoV-2 infection rate (14.29%) compared to PLWH without OIs (0.59%) (P = 0.005). The two groups were similar in ARV regimens, gender, mode of HIV acquisition, CD4 count, and HIV-VL (Table 3).

### Risk factors of SARS-CoV-2 infection among PLWH

Univariate regression analysis suggested HIV and SARS-CoV-2 co-infection is associated with elder age (age≥50, OR = 8.36, 95%CI: 3.01–23.22, P = 0.001), chronic comorbidities (OR = 4.70, 95%CI: 1.42–15.58, P = 0.011) and OIs (OR = 23.05, 95%CI: 5.93–89.57, P = 0.001), and CD4 count less than 100/μL (OR = 0.19, 95%CI: 0.05–0.76, P = 0.019). Multivariable regression analysis further suggested that age (aOR = 4.50, 95%CI: 1.34–15.13, P = 0.015) and OIs (aOR = 9.59, 95%CI: 1.54–59.92, P = 0.016) were still associated with SARS-CoV-2 infection, after adjusted for gender, chronic comorbidities, mode of HIV acquisition, CD4 count, HIV-VL and ARV regimens (Table 4).

## Discussion

This study extended the existing literature by including all three forms of SARS-CoV-2 infection and investigated the risks of total SARS-CoV-2 infection among PLWH. In May 2020 (one month after the first SARS-CoV-2 pandemic was contained in China), a cross-sectional survey showed that the positive incidence of antibody for SARS-CoV-2 in Wuhan was 4.43%[10], which is similar to the total SARS-CoV-2 infection incidence among HIV negative group in our study (6.49%). And our study showed that the total SARS-CoV-2 infection rate was lower among PLWH (1.63%) than HIV negative group, but PLWH has different infection forms of SARS-CoV-2 compared with the HIV negative group. The rate of COVID-19 reported in Wuhan was 0.45%[3] and was similar to that in both HIV negative group (0.48%) and PLWH (0.70%) that we surveyed. There was no difference in the rate of COVID-19 between the PLWH and HIV negative group in our study, which has been demonstrated in another study[11].

But we found that PLWH had more asymptomatic carriers than the control group. Although asymptomatic carriers were not found in the control group, the rate of asymptomatic carriers in PLWH (0.46%) was still higher than reported previous studies from Wuhan among the general population (8/61437, 0.013% in Wuchang district) and (221/158403, 0.001% in Wuhan city).[12, 13] Two reasons may have led to this phenomenon. First, immune deficiency in PLWH causes the body to clear the SARS-CoV-2 more slowly than in HIV negative patients.[14-16] In the general population, the median time for virus shedding in asymptomatic carriers was 19 days, and that in symptomatic infected persons was 14 days.[14] Among PLWH, the median time of virus shedding was 18 days in 68% of patients, but SARS-CoV-2 was still detected in 32% of patients 13–45 days after COVID-19 was diagnosed.[17] Secondly, PLWH may have weaker relevant clinical manifestation for their lower immunity when they were infected with SARS-CoV-2.[18, 19] This may have increased their chances of just being asymptomatic carriers at the initial stage of SARS-CoV-2 infection.

In our study, the unapparent infection of SARS-CoV-2 among PLWH (0.47%) was less than that in the HIV negative group (6.01%). After adjustment, there were still differences between the unapparent infection in the two groups. We considered two potential reasons that may have led to this phenomenon. B cell dysfunction appears during HIV infection resulting in impaired antibody responses to vaccines.[20] The first reason is that lower immunity of PLWH leads to insufficient antibody production than found in HIV negative people. The other reason we considered is that serum levels of antibodies descended faster in PLWH than in members of the general population. One study in Chongqing province of China with similar observation showed that antibodies decreased by more than 70% after two months in 90% of SARS-CoV-2 infectors, with a faster decrease rate especially in asymptomatic carriers compared to symptomatic persons in the general population.[14] Our former study also showed the positive conversion rate of IgG for SARS-CoV-2 was relatively lower and quickly lost in PLWH.[21]

Our study showed PLWH who had OIs are more likely to be infected with SARS-CoV-2. As is known to all, PLWH with OIs has severely impaired immunity, which means PLWH is easier to get other infections.[22] Some studies suggested that PLWH with tuberculosis infection are more susceptible to SARS-CoV-2 infection.[23, 24] However, reports about PLWH with OIs and SARS-CoV-2 infection are limited.[25] On the other hand, the common OIs in PLWH are tuberculosis, pneumocystis pneumonia, and bacterial pneumonia, all of which can cause lung damage,[26] and injure the local pulmonary immunity. It is noteworthy that, lower local pulmonary immunity could enhance SARS-CoV-2 infection in the theory. In addition, our data suggested that a lower current CD4 count contributes to a higher risk of acquiring SARS-CoV-2 infection. Thought the difference between the PLWH with SARS-CoV-2 and PLWH without SARS-CoV-2 was of marginal statistical significance, but the univariate regression analysis of risk factors for SARS-CoV-2 infection demonstrated that CD4 count 100/μL was one of the risk factors.

At early outbreak, many scholars have speculated that ARV drugs have therapeutic and preventive effects on COVID-19.[27, 28] But a study in Spain found ARV drugs could not reduce the morbidity of COVID-19 among PLWH.[29] Also in a randomized controlled open-label trial suggested that no benefit was observed with lopinavir-ritonavir in COVID-19 patients.[30] However, these subjects have focused on the efficacy of ARV drugs in the treatment or prevention of COVID-19 and did not suggest if ARV drugs can reduce the incidence of SARS-CoV-2 infection. Our study suggested the ARV drugs do not provide prophylaxis for SARS-CoV-2 infection among PLWH.

Our study has several limitations. First, our investigation was a cross-sectional study and hence may not reflect the conditions at the early stage of the SARS-CoV-2 pandemic in Wuhan. Another limitation is that the number of enrolled PLWH is limited, and whether the two important indicators (HIV-VL and CD4 count) that assess the immune status of PLWH are related to HIV co-infection remains to be further discussed. Third, our study sample is relatively small, which limited us to conduct more significant analyses. In addition, due to no asymptomatic infection was detected in the HIV negative group, we could not calculate the adjusted rate and 95% CI of asymptomatic and compare the difference between the two groups by Logistic model. Finally, although the serological antibody test had a certain false-positive rate, we tested each positive specimen again.

## Conclusions

PLWH were more likely to be asymptomatic and the seroprevalence of antibodies in PLWH is lower than in HIV positive population after infected SARS-CoV-2. Among PLWH, the elderly and those with OIs need to pay more attention to personal protection against SARS-CoV-2 infection. The ARV drugs do not provide prophylaxis for SARS-CoV-2 infection among PLWH.

## Figures and Tables

**Table 1:** SARS-CoV-2 infection between HIV positive and negative group in Wuchang District, 2020 (N = 1905)

	HIV-positive group (n = 857)	HIV-negative group (n = 1048)	P-value
**Age, year (means ± SD)**	39.7 ± 14.1	47.4 ± 14.2	0.001
**Gender (%)**			0.001
Male	774 (90.32)	451 (43.03)	
Female	83 (9.68)	597 (56.97)	
**Chronic Comorbidities (%)**	51 (5.95)	255 (24.33)	0.001
**Total SARS-CoV-2 infection (%)**	14 (1.63)	68 (6.49)	0.001
COVID-19	6 (0.70)	5 (0.48)	0.068
Asymptomatic Carriers	4 (0.47)	0 (0.00)	0.040
Unapparent Infectors	4 (0.47)	63 (6.01)	0.001
IgM (+) IgG (−)	1 (0.12)	11 (1.05)	
IgM (−) IgG (+)	2 (0.23)	29 (2.77)	
IgM (+) IgG (+)	1(0.12)	23 (2.19)	

**Table 2:** Comparison of SARS-CoV-2 infection between PLWH and HIV negative group in Wuchang District (N=1905)

	PLWH (n=857)	HIV negative group (n=1048)	P value
**Total SARS-CoV-2 infection**			
Crude rate (%, 95%CI) ^#^	1.63(0.78-2.48)	6.49(4.99-7.98)	
Adjusted rate (%, 95%CI) *	1.96(0.90-3.01)	5.74(4.31-7.17)	0.001
**COVID-19**			
Crude rate (%, 95%CI) ^#^	0.70(0.14-1.26)	0.48(0.06-0.90)	
Adjusted rate (%, 95%CI) *	1.10(0.11-2.10)	0.37(0.04-0.69)	0.107
**Asymptomatic carriers**			
Crude rate (%, 95%CI) ^#^	0.47(0-0.92)	0	
Adjusted rate (%, 95%CI) *	NA	NA	NA
**Unapparent infectors**			
Crude rate (%, 95%CI) ^#^	0.47(0-0.92)	6.01(4.57-7.45)	
Adjusted rate (%, 95%CI) *	0.54(0.00-1.07)	5.46(4.02-6.91)	0.001

# Confidence intervals estimated using exact binomial distribution.

* The adjusted rate was obtained after adjusting for age, gender, and chronic comorbidities using logistic regression.

**Table 3:** Demographic features of enrolled PLWH in Wuhan, China, 2020 (N = 857)

Characteristics	Uninfected SARS-CoV-2 (n = 843)	Infected SARS-CoV-2 (n = 14)	P-value
**Age (median interquartile range)**	35.0 (29.00-49.00)	53.5 (42.25-61.00)	0.005
**Gender (%)**			1.000
Male	761 (90.27)	13 (92.86)	
Female	82 (9.73)	1 (7.14)	
**Chronic Comorbidities (%)**	49 (5.81)	3 (21.43)	0.048
**Mode of HIV acquisition (%)**			
Heterosexual transmission	196 (23.25)	3 (21.43)	1.000
Homosexual	637 (75.56)	11 (78.57)	1.000
Other	10 (1.19)	0 (0.00)	1.000
**OIs (%)**			0.005
Yes	5 (0.59)	2 (14.29)	
No	838 (99.41)	12 (85.71)	
**CD4 count (cells/μL)**			0.074
<100	26 (3.08%)	2 (14.29%)	
≥100	817 (96.92%)	12 (85.71%)	
**HIV-VL (copies/mL)**			1.000
<20	618 (73.31%)	10 (71.43%)	
≥20	225 (26.69%)	4 (28.57%)	
**ARV regimens (%)**			
NRTI + NNRTI	699 (82.92)	13 (92.86)	0.485
PIs-based	78 (9.25)	0 (0.00)	0.629
INIs-based	51 (6.05)	0 (0.00)	0.587
Not on ARV	15 (1.78)	1 (7.14)	1.000

NRTI: nucleoside reverse transcriptase inhibitors. NNRTI: non-nucleoside reverse transcriptase inhibitors. PIs: protease inhibitors. INIs: integrase inhibitors. OIs: opportunistic infections.

**Table 4:** The risk factors of SARS-CoV-2 infection among PLWH, in Wuhan, China, 2020 (N = 857)

Characteristics	Univariate analysis		Multivariable analysis*	
	OR (95%CI)	P-value	Adjusted OR (aOR) (95%CI)	P-value
**Age (years)**				
18–49	1.00		1.00	
≥50	8.36 (3.01–23.22)	0.001	4.50 (1.34–15.13)	0.015
**Gender**				
Male	1.00		1.00	
Female	0.65 (0.12–3.50)	0.616	0.82 (0.07–9.12)	0.872
**Chronic Comorbidities**				
No	1.00		1.00	
Yes	4.70 (1.42–15.58)	0.011	2.17 (0.52–9.12)	0.290
**Mode of HIV acquisition**				
Non-MSM	1.00		1.00	
MSM	0.36 (0.11–1.19)	0.093	0.53 (0.13–2.11)	0.617
**OIs**				
No	1.00		1.00	
Yes	23.05 (5.93–89.57)	0.001	9.59 (1.54–59.92)	0.016
**CD4 count (cells/μL)**				
<100	1.00		1.00	
≥100	0.19 (0.05–0.76)	0.019	0.27 (0.04–1.96)	0.197
**HIV-VL (copies/mL)**				
<20	1.00		1.00	
≥ 20	1.38 (0.46–4.17)	0.567	0.98 (0.26–3.79)	0.985
**ARV regimens**				
Yes	1.00		1.00	
No	0.66 (0.04-11.00)	0.769	1.00 (1.00–1.00)	

MSM: men who have sex with men.

* Each association was mutually adjusted for the other characteristics in the table.

## Abbreviations

PLWH: people living with HIV.
SARS-CoV-2: severe acute respiratory syndrome coronavirus 2.
COVID-19: corona virus disease 2019.
HIV: human immunodeficiency virus.
AIDS: acquired immune deficiency syndrome.
aOR: after adjust rate.
95% CI: 95% confidence interval.
CDC: disease control and prevention.
CRIMS: China national HIV/AIDS comprehensive response information management system.
PSUs: primary sampling units.
ARV: antiretroviral.
OIs: opportunistic infections.
DHHS: health and human services.
CD4 count: CD4 + T lymphocyte count.
HIV-VL: HIV viral load.
RT-PCR: real-time fluorescence polymerase chain reaction.
IgM: immune globulin M.
IgG: immune globulin G.
FDA: food and drug administration.
SD: standard deviation.
